# Critical Angiographic and Sonographic Analysis of Intra Aneurysmal and Downstream Hemodynamic Changes After Flow Diversion

**DOI:** 10.3389/fneur.2022.813101

**Published:** 2022-03-10

**Authors:** Radoslav Raychev, Stanimir Sirakov, Alexander Sirakov, Hamidreza Saber, Fernando Vinuela, Reza Jahan, May Nour, Viktor Szeder, Geoffrey Colby, Gary Duckwiler, Satoshi Tateshima

**Affiliations:** ^1^Department of Neurology, University of California, Los Angeles, Los Angeles, CA, United States; ^2^Department of Radiology, University Hospital St. Ivan Rilski, Sofia, Bulgaria; ^3^Department of Radiology, University of California, Los Angeles, Los Angeles, CA, United States; ^4^Department of Neurosurgery, University of California, Los Angeles, Los Angeles, CA, United States

**Keywords:** cerebral aneurysm, flow diversion, cerebral hemodynamics, cerebral angiogram, cerebral embolization, transcranial doppler

## Abstract

**Introduction:**

Successful treatment of intracranial aneurysms after flow diversion (FD) is dependent on the flow modulating effect of the device. We aimed to investigate the intra-aneurysmal and parent vessel hemodynamic changes, as well as the incidence of silent emboli following treatment with various FD devices.

**Methods:**

We evaluated the appearance of the eclipse sign in nine distinct phases of cerebral angiography before and immediately after FD placement in correlation with aneurysm occlusion. Angiographic and clinical data of consecutive procedures were analyzed retrospectively. Patients who had successful FD procedure without adjunctive coiling, visible eclipse sign on post embolization angiography, and reliable follow-up angiographic data were included in the analysis. Detailed analysis of hemodynamic data from transcranial doppler after FD was performed in selected patients, such as monitoring for silent emboli.

**Results:**

Among all patients (*N* = 65) who met inclusion criteria, complete aneurysm occlusion at 12 months was achieved in 89% (58/65). Eclipse sign prior to FD was observed in 42% (27/65) with unchanged appearance in 4.6% (3/65) of the treated patients. None of these three patients achieved complete aneurysm occlusion. Among all analyzed variables, such as aneurysm size, device type used, age, and appearance of the eclipse sign pre- and post-FD, the most reliable predictor of permanent aneurysm occlusion at 12 months was earlier, prolonged, and sustained eclipse sign visibility in more than three angiographic phases in comparison to the baseline (*p* < 0.001). Elevation in flow velocities within the ipsilateral vascular territory was noted in 70% (9/13), and bilaterally in 54% (7/13) of the treated patients. None of the patients had silent emboli.

**Conclusions:**

Intra-aneurysmal and parent vessel hemodynamic changes after FD can be reliably assessed by the cerebral angiography and transcranial doppler with important implications for the prediction of successful treatment.

## Introduction

Flow diversion (FD) has emerged as **one** of the main methodologies for treatment of wide neck aneurysms, aimed to achieve aneurysm thrombosis by the reduction of intra-aneurysmal flow. Multiple studies utilizing various quantification methods, such as computation fluid dynamics (CFD) (1), optical flow maps (2), and angiographic parameters (3) have defined intra aneurysmal hemodynamic changes as the principal physiologic mechanism associated with treatment success after placement of FD stents. However, to this date, no specific qualitative angiographic parameters following FD have been established as a uniform predictor of successful aneurysm thrombosis. The “eclipse” sign is a characteristic angiographic finding of contrast stagnation in large and giant aneurysms after FD, historically associated with successful aneurysm thrombosis. The eclipse sign was **first** described by Lylyk et al. in the setting of FD with Pipeline Embolization Device (PED) (4). The authors demonstrated cases of contrast layering within the dependent portion of larger aneurysms typically persisting through the venous phase after embolization with PED and likely related to the marked disruption of aneurysm inflow. The appearance of eclipse sign was postulated to predict the progression to complete angiographic occlusion of the treated aneurysms. However, this hypothesis has not been fully investigated.

Another important physiologic factor associated with FD treatment of intracranial aneurysm that remains poorly investigated is the downstream hemodynamic effect on the parent vessel. The alteration of distal hemodynamics may play a pivotal role in the pathophysiology of intraparenchymal hemorrhage (IPH) and hyper-perfusion syndrome after endovascular and surgical treatment of large intracranial aneurysms (5, 6). Furthermore, the periprocedural thromboembolic risk remains a serious concern as it has been reported to occur in nearly 50% of the cases (7), yet the exact timing of this unfavorable event remains unclear. Transcranial doppler (TCD) is a readily available and easily accessible modality for bedside evaluation of intracranial hemodynamics and detection of silent emboli.

In this study, we investigated all aforementioned factors, such as qualitative and semi-quantitative comparative assessment of intra-aneurysmal hemodynamic changes by detailed appraisal of the eclipse sign in different phases of cerebral angiography, as well as the effect on downstream hemodynamics and the risk of silent emboli by critical evaluation of available TCD data in patients who underwent FD.

## Materials and Methods

This was a multi-center retrospective analysis of 63 patients who underwent 65 embolization procedures with various FD devices. Patients were included in the study if (a) the FD procedure was completed successfully without adjunctive or prior coiling, (b) there was visible eclipse sign on post embolization angiography, (c) reliable follow-up diagnostic cerebral angiogram (DSA) data were available within 12 months after initial treatment. TCD was obtained in 13 patients. The approval of institutional review board (IRB) to conduct the study was obtained at each institution. A prospectively collected database of 133 patients who underwent FD was reviewed retrospectively. All patients who did not meet the above indicated study criteria were excluded from the analyses (adjunctive/prior coiling = 44; absence of eclipse sign = 24). Pertinent clinical and procedural characteristics, such as age, gender, aneurysm size and location, type of device used, periprocedural complications, and long-term clinical and angiographic outcome were collected through retrospective review of prospectively collected data.

### Comparative Angiographic Assessment of Intra-Aneurysmal Flow Changes Using the Eclipse Sign as a Marker for Flow Stagnation Pre- and Post-FD Treatment

We evaluated all angiographic data before and immediately after FD treatment for eclipse sign appearance in nine distinct phases of cerebral angiography as follows: (1) early arterial, (2) mid arterial, (3) late arterial, (4) early capillary, (5) mid capillary, (6) late capillary, (7) early venous, (8) mid venous, and (9) late venous. All patients underwent follow-up cerebral angiography at 12 months. Retrospective evaluation of all angiographic data was conducted by two experienced neurointerventionalists, followed by consensus adjudication. Complete occlusion was defined as no residual filling (D) according to the O'Kelly–Marotta scale (8). We conducted the univariate analysis comparing five separate angiographic eclipse sign appearance patterns (presence pretreatment, presence posttreatment, unchanged appearance, earlier appearance, and prolonged appearance in more than three phases) with complete aneurysm thrombosis at 12 months, followed by multivariate regression analysis including pertinent baseline variables (age, aneurysm size, device type used, and separate angiographic eclipse sign patterns) to identify the most significant predictor of complete aneurysm occlusion at 12 months.

### Transcranial Doppler Evaluation

Complete TCD evaluation was conducted in 13 consecutive patients within 24 h post FD, of whom three patients also had pretreatment TCD. All post procedural TCD examinations were performed at the bedside in the intensive care unit (ICU). The average systolic blood pressure (SBP) was closely monitored, and it ranged between 100 and 140 during examination. Mean flow velocities and pulsatility indices (PIs) were evaluated in all vascular territories of the treated aneurysm. All 13 patients underwent 30 min of continuous monitoring of the bilateral middle cerebral artery (MCA) (11/13) and bilateral posterior cerebral artery (PCA) (2/13) territories for silent emboli. Mean flow velocities (MFV) exceeding 70 cm/s in the middle cerebral artery (MCA) and 50 cm/s in the posterior cerebral artery (PCA), and PIs ≥ 1.2 were considered increased (9, 10).

## Results

Among all patients who met inclusion criteria, 25% (16/65) were men, the mean age was 55 (±15.6), and the average aneurysm size was 16.3 (±8.17) ranging from 4 to 38 mm (Table 1). The most used FD device was P64 (Phenox, Bochum, Germany), followed by PED (Medtronic Neurovascular, Dublin, Ireland), Surpass Evolve (Stryker Neurovascular, Kalamazoo, MI, USA, and Fred (Microvention, Aliso Viejo, CA, USA). Combined ischemic and hemorrhagic complication rate was 6%, with 0% mortality, and 3% (2/65) morbidity due to delayed aneurysm rupture. About 89% (58/65) of the treated patients achieved complete aneurysm occlusion at 12 months.

**Table 1:** Clinical and procedural characteristics.

**Clinical and procedural characteristics**	
Male %	25% (16/65)
Age, mean (±SD)	55 (±15.6)
Aneurysm size in mm, mean (±SD), median (range)	16.3 (±8.17) 14 (4–38)
Device type–PED^*^	33.8% (2/65)
Device type–P64	58.5% (38/65)
Device type–Surpass Evolve	4.6% (3/65)
Device type–Fred	30% (2/65)
Occlusion rate at 12 months	89% (58/65)
Eclipse sign present prior to FD	42% (27/65)
New Eclipse sign after FD	58% (38/65)
Unchanged Eclipse Sign appearance after FD	4.6% (3/65)
Prolonged Eclipse Sign appearance in ≥ 3 angiographic phases post FD	89% (58/65)
Delayed Rupture	3% (2/65)
Thromboembolic complications	6% (4/65)
Intraparenchymal hematoma	3% (2/65)
Mortality	0%
Morbidity affecting mRS at 90 days	3% (2/65)

* *PED, Pipeline embolization device; FD, flow diversion*.

Eclipse sign prior to FD was observed in 42% (27/65) with unchanged appearance in 4.6% (3/65) of the treated patients. None of these three patients achieved complete aneurysm occlusion. *De novo* eclipse sign appearance post FD implantation was noted in 58% (38/65) of the treated patients with only limited appearance in <3 angiographic phases in four patients. None of these patients achieved complete aneurysm occlusion at 12 months.

**Figure 1:**
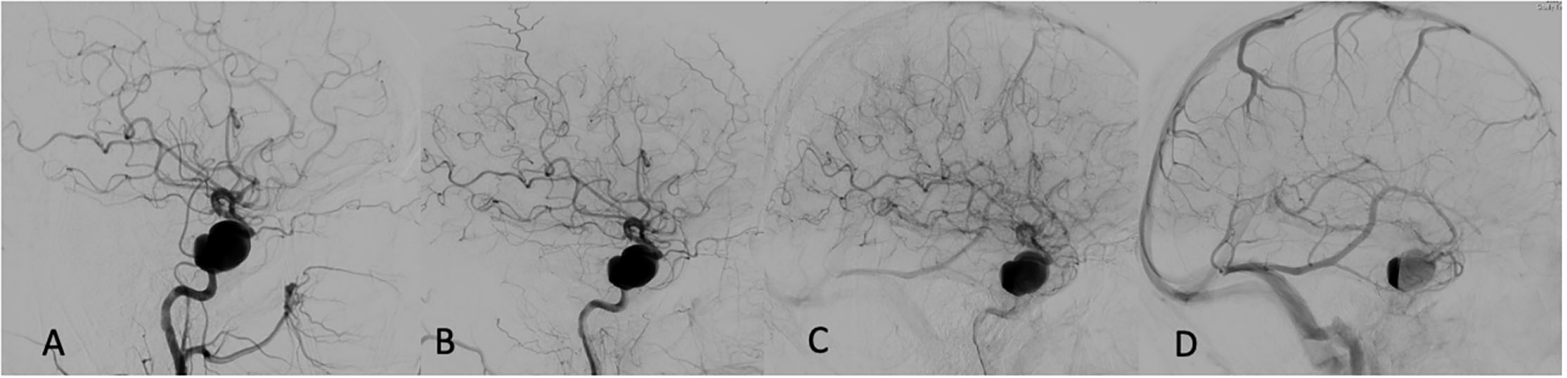
Pre-embolization angiogram: **(A)** Early arterial phase. **(B)** Late arterial phase. **(C)** Capillary phase. **(D)** Venous phase—first appearance of eclipse sign prior to flow diversion (FD).

Multivariate regression analysis revealed that among all analyzed variables, such as aneurysm size (*p* = 0.99), device type used (*p* = 0.69), age (*p* = 0.87), appearance of the eclipse sign pre-and post-FD (0.93), the most reliable predictor of permanent aneurysm occlusion at 12 months was prolonged and sustained eclipse sign visibility in more than three angiographic phases (*p* < 0.001). An example of a patient with a successfully treated large aneurysm and visible pre-intervention eclipse sign with significantly prolonged and delayed appearance post-FD embolization is depicted in Figure 1.

**Figure 2:**
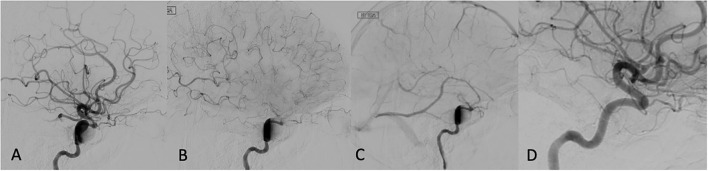
Immediate post FD angiogram: first appearance of eclipse sign in late arterial phase **(A)** persisting through capillary **(B)**, and venous phases **(C)**. A 12-months follow-up showing complete aneurysm disappearance **(D)**.

Among the 13 patients who underwent TCD evaluation, 11 had FD treatment in the anterior circulation aneurysm (Table 2). Elevation in MFV within the ipsilateral vascular territory was noted in 70% (9/13), and bilaterally in 54% (7/13) of the treated patients. All patients who had bilaterally increased MFVs also had robust flow into the contralateral MCA across the anterior communicating artery (A-Comm). Interval elevation in the PIs within the ipsilateral vascular territory was noted in 2/3 patients who underwent pre- and post-FD treatment TCD evaluation (Table 3). Detailed review of the TCD waveforms in those patients demonstrated changes in the systolic waves, accounting for the PIs elevation after FD (Figure 2). None of the patients had silent emboli or IPHs.

**Table 2:** Summary of TCD results post FD treatment.

**Aneurysm/FD device location**	**Robust contralateral flow through COW**	**MFV R MCA**	**MFV L MCA**	**PI R MCA**	**PI L MCA**
R ICA	No	**85**	50	**1.2**	**1.2**
R ICA	Yes	**75**	68	0.78	0.78
R ICA	Yes	**156**	**151**	0.82	0.8
L ICA	Yes	**90**	**101**	0.8	0.8
R ICA	Yes	**92**	**78**	0.8	1
R ICA	Yes	**159**	**101**	1	0.57
R MCA	Yes	**94**	**100**	0.57	0.73
L ICA	No	69	56	**1.2**	**1.2**
R ICA	Yes	**86**	**93**	0.9	0.82
L ICA	No	128	92	0.82	0.94
L ICA	Yes	**95**	**105**	0.8	1.1
Posterior circulation	**MFV R PCA**	**MFV L PCA**	**PI R PCA**	**PI L PCA**	
BA	No	20	38	1	1
R VA	Yes	33	30	**1.2**	0.83

*Abbreviations: COW, circle of Willis; MFV, mean flow velocity; ICA, internal carotid artery; BA, basilar artery; VA, vertebral artery; MCA, middle cerebral artery; PCA, posterior cerebral artery; PI, pulsatility index. The bold values are used to indicate abnormally elevated values*.

**Table 3:** Comparison of TCD results pre- and post-FD treatment in three patients.

**Aneurysm/FD device location**	**MCA MFV PRE**	**MCA MFV POST**	**PI RE**	**PI POST**
R ICA	82	85	0.9	1.2
R ICA	65	92	1.1	1.0
L ICA	95	93	0.9	1.2

## Discussion

Despite the proven beneficial effect and increasing utilization of FD devices for endovascular treatment of intracranial aneurysms, small percentage of the treated patients fail to achieve complete curative aneurysm obliteration and may require additional procedures (11–13). The basic mechanism of successful FD is the reduction of intra-aneurysmal flow leading to progressive thrombosis and gradual exclusion of the aneurysm from the intracranial circulation, while remodeling of the parent artery occurs around the endoluminal implant (14, 15). Following the inception of these fundamental principles and continued technological evolution, multiple FD devices with proven safety and efficacy have emerged over the past decades (16). Although seemingly different, all FD devices share a common mechanism, aimed to alter the hemodynamic interaction between the aneurysm and the parent vessel (14). Prediction of aneurysm occlusion following FD treatment is important to plan the optimal management and follow-up strategy for each individual patient. Intra-procedurally, it could help support decisions about using multiple devices to achieve the desired hemodynamic effect to maximize the aneurysm occlusion or to have closer monitoring for patients who are at a lower likelihood of aneurysm thrombosis.

As evidenced by the exhaustive literature involving CFD simulation, several key hemodynamic parameters have been identified as important predictors of successful thrombosis (17). However, despite the plethora of studies demonstrating the relationship between simulated hemodynamics and outcome, the CFD methodology remains utilized mostly in the research domain with little implications in routine clinical practice. The findings of our study provide a standardized approach for the prediction of successful FD treatment of intracranial aneurysms using readily available angiographic information. The combination of the qualitative and semi-quantitative methodology of visualization of eclipse sign presence in distinct angiographic phases used in our study is easily reproducible and widely applicable. Moreover, we established that the presence of the eclipse sign as a hallmark of aneurysmal flow stagnation can be often observed even prior to the FD treatment. In addition, we found that the presence of an eclipse sign after FD is not a reliable predictor of successful thrombosis. Instead, earlier and prolonged eclipse sign appearance is a highly predictive angiographic indicator of FD treatment success. This phenomenon can be explained by the increased intra-aneurysmal contrast residence time due to substantially decreased arterial inflow. The detailed comparative angiographic analysis between pre- and post-FD treatment presented in this study supports the aforementioned physiologic mechanisms and provides a reliable, practical, and widely applicable methodology for predicting successful aneurysm occlusion.

**Figure 3:**
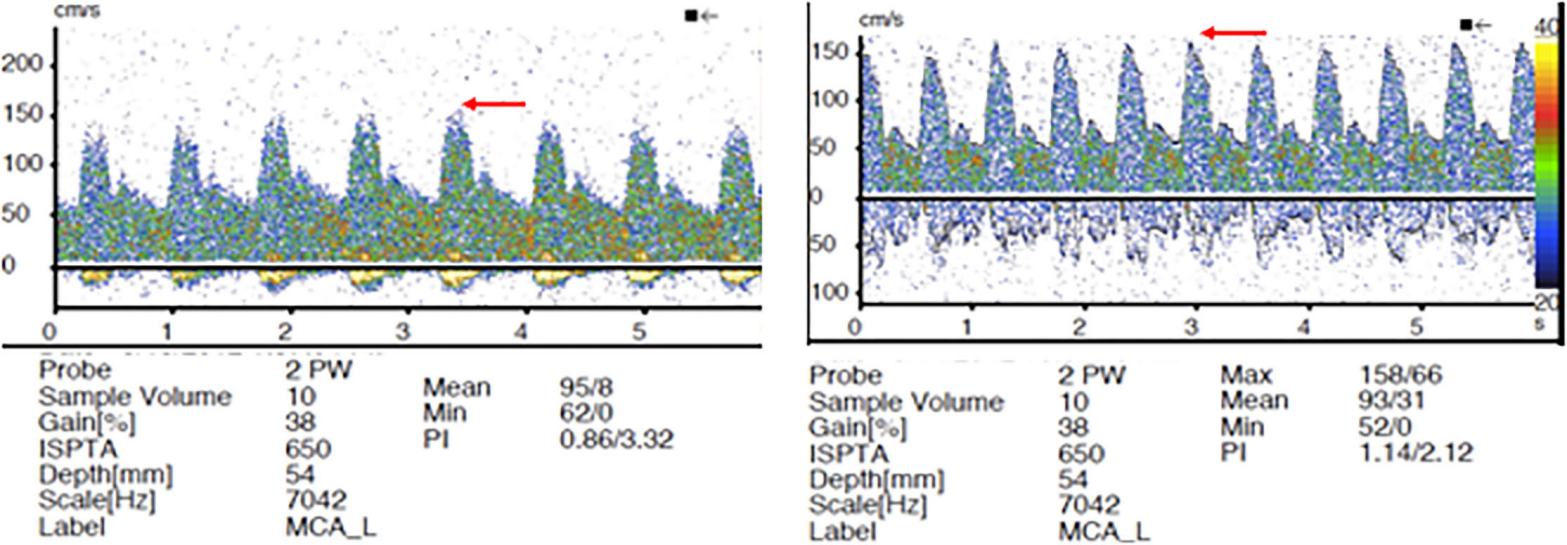
Transcranial doppler (TCD) pre- and post-FD. Elevation of pulsatility indices (PIs) post-FD device placement with more spiked appearance in the peak systolic waves (red arrows).

Aside from the significant intra-aneurysmal hemodynamic effect, it is logical to postulate that the FD treatment is associated with the downstream hemodynamic alteration. As detailed above, the well-known flow modulating effect on the aneurysm level has been a subject of extensive research. However, little is known about the parent vessel hemodynamic changes following endoluminal reconstruction with FD. Two small studies have demonstrated disrupted hemodynamics after PED placement (18, 19). The authors used quantitative magnetic resonance angiography (QMRA) to demonstrate lower MCA flows rates ipsilaterally to the treated aneurysm and attributed this phenomenon to delayed IPH—a rare and poorly understood complication of FD (20). The causal relationship between IPH and altered downstream hemodynamics was revealed by TCD examination in two patients in another small study by the same group (19). However, a substantial limitation of this study is that all TCD examinations after FD were performed under general anesthesia immediately post-procedure. General anesthesia is associated with significant alterations of cerebral blood flow (CBF) through multiple physiologic mechanisms, such as the direct effect of anesthetic agents and ventilation parameters/end-tidal pCO_2_ on cerebral vasomotor reactivity (VMR) (21). All TCD evaluations in our study were performed in an awake- and resting-state without significant alteration of normal physiologic state, except for BP control. Similar to prior studies, we established elevation of mean flow velocities in the ipsilateral vascular tree post-procedure. Furthermore, we demonstrated that contralateral MFV can be increased in patients with patent circle of Willis (COW), highlighting the FD effect on all involved vascular territories and potentially accounting for previously reported contralateral IPH (22). Another important finding in our study is the changes in PIs with more prominent peaked systolic waves (Figure 3). These findings confirm the hypothesis that the FD could alter the elasticity of the stented ICA segment, subsequently changing the blood pressure waveform propagated to the distal intracranial circulation (20, 23). Thus, it is logical to conclude that IPH after FD can be attributed to several factors, such as increased downstream hemodynamics, hemorrhagic transformation of thromboembolic events during the procedure, and dual antiplatelet-related coagulopathy.

In addition to the important pathophysiologic insights involving the parent vessel hemodynamic changes after FD, the results of our study have important practical implications. TCD is an easily accessible and widely available bedside tool, used in routine clinical practice. Patients with increased MFV and PI after FD may benefit from the more strict BP control and prolonged ICU observation, minimizing the postprocedural risk of reperfusion syndrome and IPH. Furthermore, our study demonstrates that thrombotic complications and distal embolization in the setting of FD are likely intraprocedural phenomenon.

## Limitations

There are several important limitations of this study. Given the strict inclusion criteria of absence of adjunctive coiling, we excluded many patients from our database. In addition, we excluded patients with no visible eclipse sign. Thus, our findings cannot be applied in all patients treated with FD. Another limitation is the retrospective design of the study, as well as the relatively small sample size, especially in the TCD cohort.

## Conclusions

The sole presence of the eclipse sign does not correlate with subsequent aneurysm thrombosis, and it can be often observed even prior to FD. Instead, a comparative analysis of eclipse sign pre- and post-FD with earlier, prolonged, and sustained appearance in more than three angiographic phases provides a reliable prediction of aneurysm thrombosis, irrelevant of the aneurysm size, and type of device used. FD can also be associated with substantial downstream hemodynamic changes as evidenced by the frequently observed elevation of flow velocities and changes in pulsatility indices on TCD. Distal embolization after FD is a rare phenomenon. Further studies are needed to validate these results.

## Data Availability Statement

The original contributions presented in the study are included in the article/supplementary material, further inquiries can be directed to the corresponding author.

## Ethics Statement

The studies involving human participants were reviewed and approved by UCLA IRB. The patients/participants provided their written informed consent to participate in this study.

## Author Contributions

RR and ST contributed to conception and design of the study. RR, SS, and AS organized the database. HS performed the statistical analysis. RR wrote the first draft of the manuscript. SS, AS, HS, and ST wrote sections of the manuscript. All authors contributed to manuscript revision, read, and approved the submitted version.

## Conflict of Interest

The authors declare that the research was conducted in the absence of any commercial or financial relationships that could be construed as a potential conflict of interest.

## Publisher's Note

All claims expressed in this article are solely those of the authors and do not necessarily represent those of their affiliated organizations, or those of the publisher, the editors and the reviewers. Any product that may be evaluated in this article, or claim that may be made by its manufacturer, is not guaranteed or endorsed by the publisher.
